# ADL+: A Digital Toolkit for Multidomain Cognitive, Physical, and Nutritional Interventions to Prevent Cognitive Decline in Community-Dwelling Older Adults

**DOI:** 10.3390/ijerph22010042

**Published:** 2024-12-31

**Authors:** Justin Chew, Zhiwei Zeng, Toh Hsiang Benny Tan, Pamela Chew, Noorhazlina Ali, Hao Wang, Melissa Ong, Roslyn Raymond, Kalene Pek, Di Wang, Liang Zhang, Zhiqi Shen, Cyril Leung, Jing Jih Chin, Wee Shiong Lim, Chunyan Miao

**Affiliations:** 1 Department of Geriatric Medicine, Tan Tock Seng Hospital, Singapore 308433, Singapore; 2 Institute of Geriatrics and Active Aging, Tan Tock Seng Hospital, Singapore 308433, Singapore; 3 Joint NTU-UBC Research Centre of Excellence in Active Living for the Elderly (LILY), Nanyang Technological University, Singapore 639798, Singapore; 4 Department of Psychology, Tan Tock Seng Hospital, Singapore 308433, Singapore; 5 College of Computing and Data Science, Nanyang Technological University, Singapore 639798, Singapore; 6 Lee Kong Chian School of Medicine, Nanyang Technological University, Singapore 308232, Singapore

**Keywords:** cognition, artificial intelligence, dementia, prevention, multidomain intervention, technology, aging, digital

## Abstract

Background: Current research highlights the importance of addressing multiple risk factors concurrently to tackle the complex etiology of dementia. However, limited evidence exists on the efficacy of technology-driven, multidomain community-based interventions for preventing cognitive decline. Objectives: To evaluate the efficacy of ADL+, an artificial intelligence (AI)-enabled digital toolkit integrating cognitive assessments and multidomain interventions, on outcomes of cognitive function, activity levels, and quality of life in older adults at risk of cognitive decline. Adherence and usability were also evaluated. Methods: We conducted a quasi-experimental study including community-dwelling older adults aged 60 years and above without dementia, but with subjective memory complaints (AD8 score ≥ 2). Participants received a six-month intervention (app-based cognitive training, personalized nutritional, physical, and social activities recommendations) or a control group treatment (cognitive health educational package). The primary outcome was a change in neuropsychological test battery (NTB) Z-scores (NTB composite and its individual domains: attention, processing speed, memory, and executive function). Secondary outcomes were activity levels (Frenchay Activities Index, FAI), and quality of life (EQ-5D). Outcomes were assessed at the end of the intervention and three months post-intervention using linear mixed-effects models. Results: 96% of participants in the intervention and 89% in the control group completed the study. At six months, the intervention group showed a significant NTB composite score improvement (mean change: 0.086 (95% CI 0.020 to 0.15)), with a between-group difference of 0.17 (95% CI 0.071 to 0.27). Significant differences in attention, processing speed, and memory domains were observed, with benefits sustained in the processing speed domain at nine months. The control group’s FAI scores declined at six months (mean change: −1.04 (95% CI −1.83 to −0.26)), while the intervention group’s scores remained stable. The intervention group’s EQ-5D visual analogue scale (VAS) scores improved at both six and nine months, with between-group differences of 4.06 (95% CI 0.23 to 7.90) at six months and 5.12 (95% CI 0.81 to 9.43) at nine months. Adherence was high, while average usability scores were obtained. Conclusions: The ADL+ toolkit shows potential beneficial effects on cognitive function, activity levels, and quality of life for older adults at risk of cognitive decline. Findings will guide future randomized controlled trials and implementation efforts.

## 1. Introduction

Demographic trends indicate that the number of people with dementia worldwide is expected to rise [1], with significant negative consequences at both individual and healthcare system levels. To treat dementia, there is emerging evidence supporting the use of disease-modifying therapeutics, but a better understanding of the potential risks of these treatments is required, and their long-term efficacy remains unknown [2]. On the other hand, age-specific dementia incidence is decreasing in some countries [3], a trend that may be attributed to improvements in modifiable risk factors, including lifestyle changes [4]. Thus, addressing high-risk groups to increase physical, cognitive, and social activity in mid- and later life may be an effective strategy to delay the onset of cognitive impairment and dementia. In this regard, multidomain non-pharmacological interventions that simultaneously target several dementia risk factors and disease mechanisms have been developed [5].

Despite promising evidence of efficacy, implementation of multidomain interventions may be challenging due to a few factors. For instance, adherence to planned interventions is variable. In the Finnish geriatric intervention study to prevent cognitive impairment and disability (FINGER) [6], while overall adherence rates were high, only 38.9% of participants adhered to all intervention components, with the lowest levels reported for the cognitive training component [7]. Also, while the results of some multidomain interventions are positive, not all studies report significant benefits [8]. Dementia has a complex, multifactorial etiology, where inter-individual and population variability in risk factors render a ”one-size-fits-all” approach less effective. Accordingly, the European Task Force for Brain Health services suggested that multidomain intervention effectiveness may depend on a more personalized prevention approach [9].

To overcome these limitations, there has been increasing interest in leveraging technology for interventions targeting cognitive health. For example, computerized cognitive training (CCT) uses programmes or software to target specific cognitive domains, with the potential to improve global cognition, memory, attention and psychosocial functioning [10]. Other technology applications include artificial intelligence (AI) and gamification techniques to support individualized lifestyle interventions and drive positive behavioral change. For instance, AI has been used to identify patterns in dietary habits and activity levels, providing personalized nutrition and physical activity recommendations [11]. However, there is a paucity of evidence supporting this approach, and results from available studies are inconsistent [12]. More data are needed to determine the impact of technology-enabled multidomain interventions on clinical outcomes.

Therefore, the primary objective of the study was to evaluate the effectiveness of the multidomain intervention on cognitive performance as measured by standardized neuropsychological tests. The secondary objective was to examine the effects of ADL+ on outcomes of activity levels and quality of life. We hypothesized that the intervention group would perform better than the control group in the primary and secondary outcomes. We also collected adherence data and examined the usability of the ADL+ platform to provide perspectives from older persons engaging with this technology. This study will contribute to the evidence on technology-assisted multidomain physical, cognitive, nutrition, and social interventions for improving cognitive health in healthy older adults at risk of dementia.

## 2. Materials and Methods

### 2.1. Study Design and Participants

The ADL+ study is a longitudinal, quasi-experimental study, with participants recruited from nine senior activity centers in Singapore, including individuals with subjective memory complaints, assessed using the locally validated ≥2 cut-off for the AD8 interview [13]. Participants were included if they met the following criteria: age 60 years and above; the presence of subjective memory complaints (AD8 score ≥ 2); mCMMSE scores above the locally validated age- and education-specific cut-offs for dementia (>21 for 0 to 6 years of education; >24 for more than six years education) [14]; independent in activities of daily living (ADL); able to speak English and/or Mandarin; and comfortable with using smartphones. The exclusion criteria were as follows: known or suspected dementia after the initial screening, significant medical illnesses such as active malignant disease, major depression, symptomatic cardiovascular disease, severe loss of vision, hearing, or the inability to communicate.

**Figure 1:**
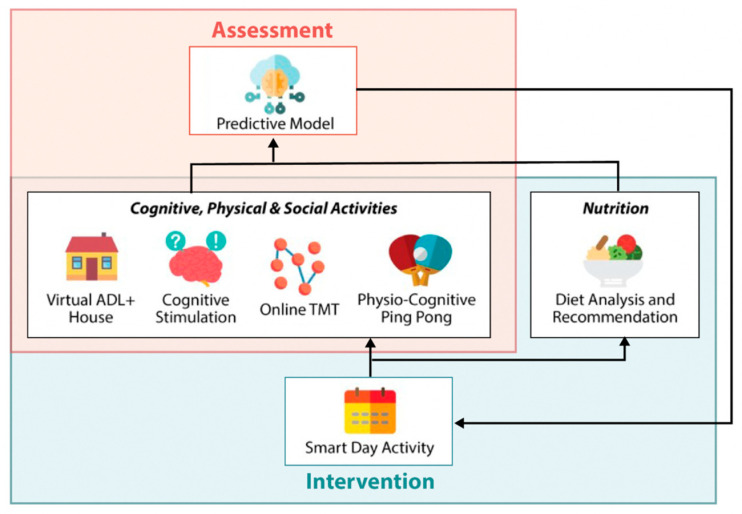
The ADL+ toolkit with assessment and intervention modules.

Participants were recruited into two arms, intervention and control (Figure 1). The intervention group participated in the ADL+ intervention for six months. The control group received printed educational materials about cognitive health and dementia, which included information on a healthy diet, physical activity, and mental and social well-being. As the study coincided with the COVID-19 pandemic, the number of centers and potential participants was limited due to restrictions imposed as part of the national public health measures [15]. Recruitment was adjusted to align with these protocols, restricting the number of participants attending centers and proceeding only when permitted. These constraints posed significant logistical challenges, necessitating adjustments to optimize recruitment, with the allocation of participants to study groups guided by practical considerations of center capacity and individual availability.

Ethics approval for this study was obtained from the National Healthcare Group (NHG) Domain Specific Review Board (Study Reference Number: 2018/01089).

### 2.2. Intervention

#### ADL+ Toolkit

The ADL+ toolkit incorporates a collection of modules designed for simultaneous cognitive monitoring and personalized intervention. These modules offer technology-enabled gamified interventions covering the areas of cognitive training, physical exercise, and diet, while also serving as cognitive monitoring tools. By leveraging the data gathered from the toolkit, such as nutritional intake and game performance, the toolkit analyzes and learns representations of an individual’s cognitive functioning. These representations are then utilized to personalize interventions according to the individual’s cognitive status. In this study, participants were instructed to use the application platform for six months.

The ADL+ toolkit consists of seven modules (Figure 1): (a) Smart Day Activity: This module serves as an intelligent system that facilitates effective navigation and coordination within the toolkit. It provides scheduling, advisory, and incentive functionalities to guide participants in their interactions with other modules. (b) Virtual ADL+ House: This module features a virtual home environment that integrates cognitive assessment and training. It incorporates gamified cognitive exercises within eight scenarios related to instrumental activities of daily living, such as cooking, financial management, laundry, medication, shopping, telephone use, and transportation. (c) Online Trail-Making Test (TMT): This module is a Kinect-based and gamified adaptation of the TMT, a well-established neuropsychological test measuring attention and executive function. (d) Physio-Cognitive Ping Pong: This Kinect-based table tennis simulation promotes physical and cognitive exercise through dual-tasking. Participants are presented with questions and are required to answer by hitting back one of two incoming balls tagged with answers, using a physical swinging motion. This module encourages both physical activity and social interaction. (e) Cognitive Stimulation: This smartphone game delivers the individual cognitive stimulation therapy (iCST) through an app-based platform. It encompasses a diverse range of themed activities based on visual and auditory puzzles, life memories, current affairs, number and word games, and object categorization. (f) Diet Analysis and Recommendation: This module is an AI-driven, image-based tool that monitors food intake, analyzes nutritional content, and promotes healthier eating habits. Using advanced computer vision, it identifies food items from meal photos and estimates their nutritional values. To improve recognition of Singapore’s diverse local cuisines, a dataset of 500+ dishes and 140,000 images was developed. The system employs a deep convolutional neural network (CNN) model, which is linked to a specialized nutrition database from the local Health Promotion Board. The system automatically retrieves and updates nutritional values, allowing user adjustments, and generating insights comparing intake to recommended daily values, thus helping users make healthier dietary choices. (g) Predictive Model: This model utilizes data collected from other modules, such as nutrition intake and game performance data, to perform data analytics and assess an individual’s cognitive functioning.

The Smart Day Activity app serves as the primary interface for participants within the ADL+ toolkit. Each day, the participants receive a personalized task schedule generated by the Smart Day Activity app. The Cognitive Stimulation module and the Diet Analysis and Recommendation Module are integrated within the Smart Day Activity app. The Cognitive Stimulation activities are scheduled daily, with a single theme assigned each time. Diet logging is scheduled three times daily, corresponding to breakfast, lunch, and dinner, with an option for participants to input additional entries for snacks consumed. The Smart Day Activity app also recommends other creative activities to participants, including drawing, singing, and origami folding, twice or thrice a week. Virtual ADL+ House, Online TMT, and Physio-Cognitive Ping Pong are community-based activities that take place at designated senior activity centers, with iPads and Kinect systems made available at these centers for participants to utilize. These modules are scheduled weekly, taking into account the preferences of both the participating center and the individual participant. Each participant is assigned two out of the three community activities per week. Prior to the scheduled community activities, participants receive notifications through the Smart Day Activity app. Upon arrival at the community center, they can conveniently log in and access their assigned activities using a personalized in-app QR code. Detailed descriptions of the ADL+ toolkit and screenshots are available in Supplementary Materials S1.

### 2.3. Measures

#### 2.3.1. Outcomes

Cognitive assessments were performed using a standardized neuropsychological test battery (NTB) at baseline, six months, and nine months (three months post-intervention), conducted by trained assessors supervised by a clinical psychologist experienced in conducting neuropsychological tests. The primary outcome was a change in cognitive test performance, as measured by an NTB composite comprising the following domains: attention, processing speed, memory, and executive function. Neuropsychological test Z-scores were standardized to the baseline mean and standard deviation (SD), with higher scores indicating better performance. The neuropsychological battery included the following: attention domain (Colour trails test 1 [16]); processing speed (Symbol Search [17] and Symbol Digits Modalities Test [18]); memory (Logical Memory sub-test from the Wechsler Memory Scales—4th Edition (WMS-IV) [19]) and executive function (Colour trails test 2 [16] and animal category [20]). NTB composite and domain-specific scores were obtained for analysis. In addition, a global measure of cognitive function was also used, using the mCMMSE [14].

The secondary outcomes included activity levels, measured on the Frenchay Activities Index (FAI) [21] and health-related quality of life (QoL). We assessed QoL in two ways: (1) using index scores of the five-level version of the EuroQol five-dimensional (EQ-5D-5L) questionnaire [22,23] based on Singapore preference weights derived using an indirect interim mapping method; [24,25] and (2) self-rated health on the EQ-5D visual analogue scale (VAS).

#### 2.3.2. Adherence and Usability

We evaluated overall adherence to the ADL+ toolkit by determining the number of days participants completed all required activities. Criteria for completion were as follows: for days with assigned community activities (2 mini-games from ADL+ House, Online TMT, or Ping Pong), participants had to complete the 2 community activities as well as any assigned EQ-5D-5L questions and weight measurements. For days without community activities, participants needed to complete iCST, submit 3 diet logs, and finish any assigned EQ-5D-5L questions and weight measurements. Additionally, we assessed adherence to each individual module (Virtual ADL+ House, iCST, Online TMT, Physio-Cognitive Ping Pong, and Smart Day Activity Recommendations) by calculating the percentage of completed sessions relative to the total number of assigned sessions for each module. Adherence to the Diet Analysis and Recommendations modules was determined by the percentage of times participants logged their daily dietary intake as scheduled.

After the intervention, we assessed user experience by employing the System Usability Scale (SUS) to gather quantitative data [26]. The SUS is a widely accepted and psychometrically reliable instrument used for evaluating users’ perceptions of the usability of technological systems [27]. The SUS consists of 10 items, each rated on a 5-point Likert scale (1 = “strongly disagree” to 5 = “strongly agree”). Using the standardized scoring protocol, we derived a score ranging from 0 to 100, with higher scores denoting better usability. A widely accepted benchmark for SUS scores is a score of 68 [28], which denotes an average level of usability. Additionally, we collected qualitative feedback through open-ended questions, which inquired about participant preferences for specific modules and the rationale behind their choices.

### 2.4. Statistical Analysis

#### 2.4.1. Sample Size Calculation

We based our sample size calculations on the primary outcome of change in the NTB total score. With a 5% significance level and 90% power, the required sample size was estimated to be 62 subjects per arm to detect a difference of 0.022 (SD = 0.5) in composite NTB Z-score between groups [6]. Accounting for a dropout rate of 20%, the projected sample size would be 74 per group (or a total of 148 participants).

#### 2.4.2. Effectiveness

To determine the intervention effects of ADL+, skewed NTB components were first log-transformed. Z-score differences from baseline were evaluated at 6- and 9-month follow-ups for the neuropsychological test outcomes and raw scores for mCMMSE, FAI, EQ-5D-5L utility scores, and VAS. Differences from the baseline were estimated using repeated-measures linear mixed-effects models. In addition to the trial group (control versus intervention), time, and the interaction of the trial group with time, the model included the respective baseline outcome scores, age, gender, education level, and baseline Geriatric Depression Scale (GDS) [29] scores as covariates.

Statistical significance was set at *p* < 0.05. Stata statistical software (version 15) was used for all analyses.

#### 2.4.3. Adherence and Usability

We presented adherence data as medians and interquartile ranges for both the overall intervention and individual modules. For usability data, we calculated the mean and standard deviation for both the total SUS score and individual item scores. For the open-ended survey, questions were posed in either English or Mandarin to facilitate accurate responses. For Mandarin-speaking participants, a bilingual research assistant transcribed their responses from Mandarin to written English. All non-Chinese participants provided their responses in English. We conducted thematic analysis of the qualitative feedback using the Braun and Clarke framework [30].

## 3. Results

**Figure 2:**
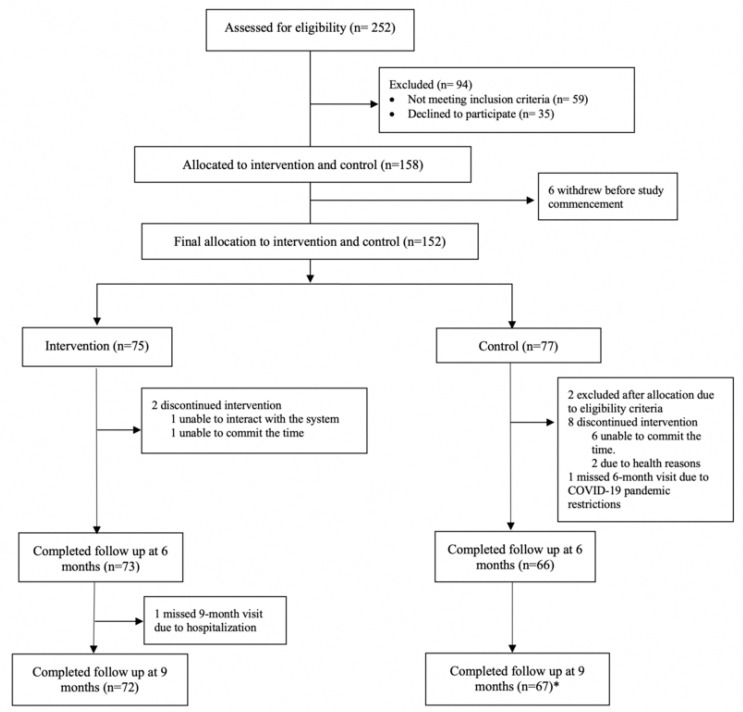
Study flow chart. * Inclusive of participant who missed the 6-month visit due to COVID-19 pandemic restrictions.

Recruitment took place between September 2019 and December 2020, with 252 screened and 150 assigned to the intervention (*n* = 75) and control group (*n* = 75). A total of 142 (93%) participants completed the nine-month assessments, with three dropouts in the intervention group and seven in the control group (Figure 2). There were no differences in demographic characteristics, education levels, or cognitive and depressive symptoms between dropouts and participants who completed the study.

Baseline characteristics are shown in Table 1. Participants in the control group were older and had lower education levels, poorer cognitive scores, and higher depressive symptoms compared to the intervention group. There were no other significant differences in cardiovascular comorbidities except for a higher proportion of individuals with hyperlipidemia in the control group.

### 3.1. Cognitive Outcomes

**Figure 3:**
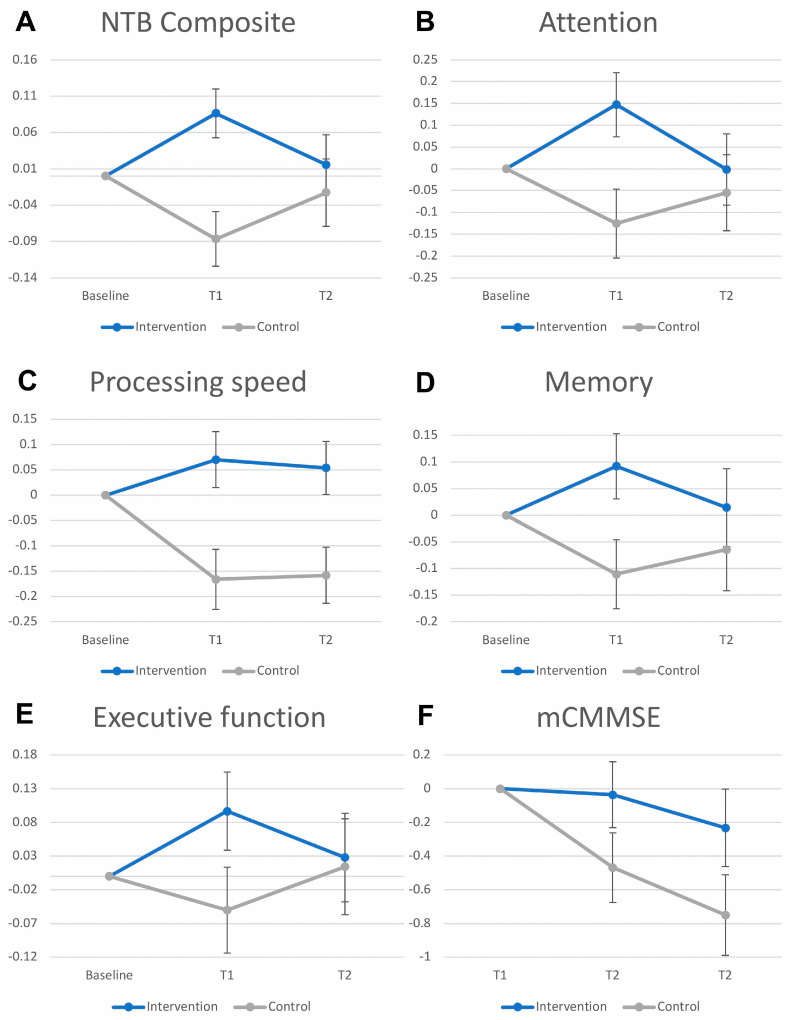
Cognitive, activity levels, and quality of life outcomes of the ADL+ intervention.

Results of the neuropsychological outcomes are shown in Figure 3 and in Supplementary Materials Table S2. At six months, the intervention group showed a mean increase of 0.086 (95% CI 0.020 to 0.15) on the NTB composite from baseline, whereas the control group had a decrease of −0.087 (95% CI −0.16 to −0.013) from baseline, for an estimated mean difference between intervention and control of 0.17 (95% CI 0.071 to 0.27, *p* = 0.001). For individual cognitive domains, statistically significant mean differences between groups were observed for the attention, processing speed, and memory domains at six months, but not for executive function.

At nine months, intervention effects were sustained only in the processing speed domain. A non-statistically significant mean change in processing speed scores of 0.054 (95% CI −0.049 to 0.16) was observed in the intervention group, whereas the control group showed a significant decrease of −0.16 (95% CI −0.27 to −0.050), for an estimated mean difference between intervention and control of 0.21 (95% CI 0.059 to 0.37). The intervention effects were no longer sustained at nine months for the NTB composite score, nor in the attention, memory, and executive function domains.

For mCMMSE scores, mean differences between intervention and control groups at 6 and 9 months were non-significant. However, control group participants experienced statistically significant decreases in mCMMSE scores from baseline at both time points (estimated mean change at six months: −0.47 (95% CI −0.87 to −0.063); estimated mean change at nine months: −0.75 (95% CI −1.22 to −0.28)), whereas no significant changes were observed for the intervention group across both time points.

### 3.2. Activity Levels

Results of the secondary outcomes are shown in Figure 3 and in Supplementary Materials Table S2. For activity levels measured by the FAI at six months, a non-statistically significant mean change of 0.40 (95% CI −0.35 to 1.15) was observed in the intervention group, whereas the control group showed a significant decrease of −1.04 (95% CI −1.83 to −0.26), for a mean difference of 1.44 (95% CI 0.33 to 2.56, *p* = 0.011). At nine months, FAI scores in both the intervention and control groups declined from baseline, with no significant between-group differences (*p* = 0.32).

### 3.3. Quality of Life

Regarding quality of life, measured using EQ-5D-5L utility scores, no significant changes were observed between intervention and control groups at 6 and 9 months (Figure 3 and Supplementary Materials Table S2). However, the intervention group showed significant improvements in EQ-5D-5L VAS scores at both time points compared to baseline (6 months: 4.46 (95% CI 1.89 to 7.04); 9 months: 5.46 (95% CI 2.52 to 8.40)). The control group did not exhibit significant changes in EQ-5D-5L VAS scores across both time points. The mean difference in EQ-5D-5L VAS scores between the intervention and control groups was 4.06 (95% CI 0.23 to 7.90, *p* = 0.038) at six months, and this effect persisted at nine months (mean difference: 5.12 (95% CI 0.81 to 9.43, *p* = 0.020)).

### 3.4. Adherence and Usability

Adherence data were collected for all 75 participants in the intervention group. The overall median adherence rate for the ADL+ toolkit was 95% (IQR 88.3–98.3%), and adherence was 90% and above for each individual ADL+ module (Supplementary Materials, Table S3).

For the usability analysis, the total and mean scores for each of the 10 items in the SUS were calculated and reported in Table 2. The ADL+ platform achieved a mean (SD) SUS score of 70.7 (7.1) out of 100, indicating an average usability score. The scores ranged from 3.48 to 4.04 on a scale of 1 to 5, with higher scores indicating better usability. The highest mean scores were obtained for item 3 (“I thought the system was easy to use”) and item 9 “I felt very confident using the platform” at 4.00 and 4.04, respectively. The lowest mean score (reverse-coded) was obtained for item 10 (“I needed to learn a lot of things before I could get going with this platform”), which reflects the perception of a steeper learning curve in learning to use the platform during the initial stages.

The most-liked components of the ADL+ toolkit were the Telephone mini-game within the ADL+ House (*N* = 27), Online TMT (*N* = 24), and Physio-Cognitive Ping Pong (*N* = 22). We identified three themes which explicated why participants liked these modules: cognitive stimulation; level of engagement; and user experience and motivation. Participants opined that these games were progressively difficult and “challenging enough” for them, and trained different domains of cognition such as memory, attention, and processing speed. Pertaining to the level of engagement provided by the games, participants regarded them as interesting and fun, with interactive opportunities to “move around and exercise”. In terms of user experience, participants were “encouraged by the point system” that provided instant feedback on their progress. The ease of learning further facilitated technological adoption, with some participants being “less fearful of technology”.

The least-liked elements of the ADL+ toolkit included the Transport mini-game within the Virtual ADL+ House module (*N* = 15) and the iCST (*N* = 7). The identified themes related to these less-favored components involved cognitive load, perceived lack of stimulation, and platform usability. Concerning cognitive load, some games were perceived to be “too difficult” beyond specific levels, with “too many numbers” and “too many things” to recall. In contrast, some participants perceived the games to lack stimulation by virtue of being “too easy” or having questions which are “repetitive”. Lastly, participants raised issues relating to technical problems, unclear instructions, and the small size of graphic elements.

## 4. Discussion

The current study evaluates the efficacy of ADL+, a multidomain intervention targeting older adults at risk of cognitive decline. The intervention incorporated AI-enabled data analytics, behavioral prompts to increase cognitive and physical activity, diet tracking and analysis, and social interaction through a mobile platform and center-based components. ADL+ had a significant beneficial effect on overall cognition, as measured by neuropsychological composite scores and a global measure of cognition. Specific cognitive domains showing benefits include the memory, processing speed, and attention domains. Intervention effects were sustained for processing speed at three months post-intervention. Participants in the intervention group maintained their overall activity levels and improved self-reported health-related quality of life compared to the control. Adherence to the intervention was also high, while average usability scores were obtained.

Dementia prevention trials have largely evolved beyond single to multidomain interventions, with the recognition that modifying multiple risk factors across the life course may prevent or delay up to 40% of dementia cases [4]. Multidomain interventions generally achieve improvements in cognitive domains of executive function, memory, and verbal fluency compared with single domain interventions or control [31], but other studies report inconsistent effects [32]. For instance, the FINGER trial, a 2-year multidomain intervention targeting exercise, diet, cognitive training, and vascular monitoring, demonstrated positive effects on global cognition, executive functioning and processing speed [6]. Similarly, in the 3-year Multidomain Alzheimer Preventive Trial (MAPT) which incorporated physical activity, cognitive training, and nutritional advice, a positive effect on cognitive scores was observed, albeit in exploratory subgroup analyses [33]. In comparison with previous studies [34,35], the ADL+ toolkit incorporates cognitive monitoring and AI-driven personalized intervention, which enables a user-specific experience that adapts to an individual’s progress. ADL+ also integrates home- and center-based cognitive training with social elements, offering greater accessibility and flexibility in how users engage with the ADL+ toolkit, contrasting with other setting-specific methods. Moreover, limited evidence exists on digital interventions aiming to deliver personalized care [12], with emerging evidence of positive results in terms of cognitive outcomes [36]. Our results encompass a comprehensive evaluation of cognitive, activity, and quality of life outcomes, supporting the potential of digital multidomain lifestyle interventions for prevention of cognitive decline.

In the current study, our findings were notable for the beneficial intervention effects of CCT-based cognitive stimulation and training in the attention, processing speed, and memory domains. In the published literature, meta-analyses have summarized the effects of CCT on different cognitive functions in healthy older adults and individuals with mild cognitive impairment [37,38,39]. Effects varied depending on the cognitive domain assessed, with some CCT interventions showing improvements in the memory and processing speed domains, with moderate effect sizes, but not for executive function [38,40]. Similar to our study, where improvements in processing speed were observed at six-month and also at three-month follow-ups, another meta-analysis observed that the largest effect size was observed for the processing speed domain [41], a finding of particular salience in the light of processing speed being a hallmark of cognitive aging and one of the strongest predictors of performance across cognitive tasks in older adults [42]. The Active Training in Vital Elderly (ACTIVE) trial also demonstrated that cognitive training in processing speed was associated with larger improvements in cognitive performance, wider transfer to daily function, and reduced dementia risk [43].

In addition to cognitive stimulation and training, the ADL+ toolkit was designed to promote social interaction through gamification approaches, resulting in sustained activity levels and improved self-reported quality of life. Components such as the Online Trail Making Test and Physio-Cognitive Ping Pong aim to support various aspects of well-being, including mental, physical, and social engagement. The Smart Day Activity app prompts individuals to visit their nearby active aging Centers (AACs) in Singapore, which are equipped with iPads and Kinect systems, enabling social interaction during these activities. A growing body of research supports the effectiveness and motivational potential of integrating game-like elements into technology to facilitate older adults’ engagement with health technologies [44]. Preliminary findings also indicate that gamification can boost social activity among older adults. Our study provides initial support for embedding social elements in technology-based multidomain interventions to foster social involvement among this demographic [45,46]. Furthermore, the ADL+ toolkit purposefully employs gamification techniques to encourage older adults to venture beyond their homes and devices to participate in community-based activities. Our results suggest that incorporating gamification strategies into technology-based solutions for older adults may hold promise for effectiveness.

In the current study, we integrated personalized nutrition analysis as part of a broader approach to cognitive health and dementia prevention. Although definitive evidence linking nutrition and cognitive health has yet to be established, maintaining a healthy diet is crucial for healthy aging. Good nutrition can reduce the incidence of diseases like diabetes and hypertension, which are known risk factors for dementia. The Diet Analysis module in our study, based on a local nutrition database from the Health Promotion Board, assessed dietary habits and generated summaries according to locally recommended dietary guidelines. Combined with cognitive training interventions in the ADL+ toolkit, this multidomain approach addresses key factors related to both nutrition and cognitive health in the context of dementia prevention.

A growing number of studies aim to leverage digital biomarkers and AI methods to facilitate the monitoring and prediction of cognitive decline, with the goal of implementing individualized multidomain, preventive lifestyle interventions through passive and active data collection [47]. However, further studies are still needed before accurate and reliable prediction tools can be used in clinical practice and for decision-making. ADL+ components were designed to provide data input for predictive modelling to deliver individualized recommendations across the relevant domains. Specifically, the predictive model computes analytics to predict cognitive decline and feed this information to the Smart Day Activity app to adapt the intervention programme based on individual requirements. Our study provides preliminary data for the potential efficacy of such electronic health interventions incorporating adaptive interventions [48] and personalized or AI digital coaching aimed at increasing physical activity levels and overall well-being [49].

In this study, we observed high adherence rates across all modules of the ADL+ toolkit, with an overall median adherence of 95%. The high adherence could be attributed to the combination of personalized prompts, AI-driven reminders, and the supportive role of research assistants and center staff who maintained constant contact with participants and were available to troubleshoot technical difficulties. However, it is important to note that these results were obtained in the context of a research study. Further investigations are needed to understand real-world adherence when such interventions are implemented outside a controlled research environment. Nonetheless, the consistently high adherence observed in both community-based interventions and individual modules suggests that integrating technology and human support can potentially improve adherence in interventions targeting older adults, which may lead to more meaningful and lasting outcomes.

Our qualitative data highlight both the potential benefits and challenges faced by older adults when engaging with mobile apps and games for cognitive training. Through the thematic analysis, it is evident that older adults can be receptive to technology interventions. Encouragingly, positive feedback from participants indicates that interventions like ADL+ can help reduce older adults’ apprehension towards technology and promote its adoption. However, factors such as excessive cognitive load, lack of stimulation, and platform usability emerged as critical concerns in relation to the least-liked components. Our research supports earlier investigations into barriers hindering technology adoption among older adults, with technical challenges and difficulties understanding and navigating mobile interfaces being major barriers to adoption [50]. Drawing from the tenets of the cognitive load theory [51], it is vital to consider the older adults’ capacity to handle the different types of cognitive loads: intrinsic load, which is tied to the inherent complexity of the task; extraneous load, which arises from the platform usability and design issues; and germane load, which refers to the mental effort used to process and retain relevant information. By effectively managing the intrinsic and extraneous loads, older adults can optimize germane load and maximize the benefits of ADL+. Thus, to address these factors, user interfaces must be designed to meet the distinct requirements and preferences of older adults. For instance, providing clearer instructions and options for adjustable text or graphic sizes could accommodate those with visual impairments, making the intervention more accessible and user-friendly for this population. Additionally, personalizing cognitive games to one’s individual abilities will accommodate the diverse cognitive capacities and learning preferences among individuals. By doing so, we can enhance their engagement and foster the adoption of technology-based interventions in this population.

While the present study demonstrated potential beneficial effects of the ADL+ toolkit, several limitations exist. Firstly, the non-randomized study design and the imbalance in group characteristics at baseline may have introduced bias. While we adjusted for recognized confounders in the multivariate analysis, residual bias might remain. Secondly, our study was not designed to assess transfer effects, and existing evidence on the efficacy of cognitive training for producing such effects remains inconclusive [52,53], with effects varying based on factors like intervention type, target population, and the cognitive domains assessed. Nonetheless, evidence suggests that interventions aiming to enhance processing speed and attentional control—domains that demonstrated improvements in our study—have the potential to generalize to older adults’ everyday functioning. Third, we evaluated outcomes after six months of intervention and three months post-intervention, leaving the long-term sustainability and effectiveness of these interventions unexplored. Although most cognitive domains and activity levels did not show sustained effects post-intervention, self-reported quality of life measures remained significant, indicating a possible carry-over effect. Future research could explore strategies like booster sessions or continued platform engagement to maintain consistent levels of cognitive, physical, and social activity for optimal outcomes [54]. Fourthly, our study may not be generalizable to all older adults, as it included individuals comfortable with smartphones and was conducted in the cultural context of Singapore. Other factors, such as varying educational levels, spoken language, socioeconomic status, and different levels of technology acceptance and familiarity must also be considered when interpreting our findings. Lastly, AI methods offer potential for enhancing digital health interventions, but challenges such as computational demands, data annotation, and explainability must be addressed for real-world implementation [55].

## 5. Conclusions

Our study evaluated the efficacy of an AI-enabled, app-based multidomain intervention for preventing cognitive decline in healthy community-dwelling older adults using a proof-of-concept quasi-experimental study. The beneficial effects observed on attention, memory, processing speed, and overall neuropsychological test performance suggest that a personalized approach, facilitated by technology, may effectively deliver multidomain interventions. Furthermore, our results, showing improvements in activity levels and quality of life, even amidst a pandemic, attest to this approach’s potential to drive participation and physical activity. Further studies employing a randomized controlled design and implementation research [56], with adaptations for different populations, are required to further evaluate the effectiveness and implementation of ADL+ in real-world settings.

## Figures and Tables

**Table 1:** Baseline characteristics.

	Whole Cohort (*N* = 150)	Intervention (*N* = 75)	Control (*N* = 75)	*p*
Demographic characteristics				
Age in years, mean (SD)	71.2 (5.5)	69.7 (4.8)	72.6 (5.9)	0.0012
Female gender, *N* (%)	115 (76.7)	56 (74.7)	59 (78.7)	
Ethnicity, *N* (%)				0.45
Chinese	138 (92.0)	71 (94.7)	67 (89.3)	
Malay	4 (2.7)	1 (1.3)	3 (4.0)	
Indian	8 (5.3)	3 (4.0)	5 (6.7)	
Education level in years, mean (SD)	7.8 (3.9)	8.5 (3.8)	7.0 (4.0)	0.018
Comorbidities, *N* (%)				
Hypertension	73 (48.7)	36 (48.0)	37 (49.3)	0.87
Hyperlipidemia	88 (58.7)	37 (49.3)	51 (68.0)	0.02
Diabetes mellitus	31 (20.7)	12 (16.0)	19 (25.3)	0.16
Ischemic heart disease	14 (9.3)	6 (8.0)	8 (10.7)	0.58
Myocardial infarction	5 (3.3)	3 (4.0)	2 (2.7)	0.65
Stroke or TIA	9 (6.0)	2 (2.7)	7 (9.3)	0.086
Cognitive scores				
mCMMSE, mean (SD)	25.8 (1.6)	26.1 (1.4)	25.5 (1.7)	0.0099
AD8, mean (SD)	3.2 (1.1)	2.9 (1.0)	3.5 (1.2)	0.0032
Functional and activity scores				
ADL total score, mean (SD)	97.8 (4.1)	98.2 (3.7)	97.5 (4.5)	0.28
IADL total score, mean (SD)	21.9 (1.5)	22.0 (1.3)	21.8 (1.7)	0.5
FAI total score, mean (SD)	28.5 (4.1)	29.0 (3.9)	28.1 (4.3)	0.17
Depressive symptoms				
GDS, mean (SD)	2.2 (2.3)	1.7 (1.6)	2.7 (2.8)	0.0087
Quality of life scores				
EQ-5D utility score, mean (SD)	0.89 (0.17)	0.89 (0.16)	0.89 (0.17)	0.99
EQ-5D VAS, median (IQR)	80 (70–80)	80 (70–80)	80 (65–85)	0.66

SD, standard deviation; TIA, transient ischemic attack; mCMMSE, modified version of the Chinese Mini-Mental State Examination; ADL, activities of daily living; IADL, instrumental activities of daily living; FAI, Frenchay Activities Index; GDS, Geriatric Depression Scale; EQ-5D, EuroQoL-5 Dimensions; VAS, visual analogue scale, IQR: interquartile range.

**Table 2:** System Usability Scale (SUS) item and total scores *.

	SUS Items	Mean (SD) Scores
1	I think that I would like to use this platform frequently	3.84 (0.71)
2	I think that the platform is unnecessarily complex.	3.82 (0.63)
3	I think that the platform is easy to use.	4.00 (0.24)
4	I think that I would need the support of a technical person to be able to use this platform.	3.99 (0.39)
5	I think that the various functions in this platform are well integrated.	3.96 (0.42)
6	I think that there is too much inconsistency in this platform.	3.74 (0.71)
7	I think that most people would learn to use this platform very quickly.	3.63 (0.70)
8	I think that this platform is very cumbersome to use	3.78 (0.69)
9	I felt very confident using the platform.	4.04 (0.26)
10	I needed to learn a lot of things before I could get going with this platform.	3.48 (0.94)
	SUS Total	70.7 (7.1)

* Each item is rated on a 5-point Likert scale (1 = “strongly disagree” to 5 = “strongly agree”), with even-numbered items reverse-scored for calculating the SUS total.

## Data Availability

The data presented in this study are available on request from the corresponding author due to privacy restrictions.

## Supplementary Materials

The following supporting information can be downloaded at: https://www.mdpi.com/article/10.3390/ijerph22010042/s1, Supplementary Materials S1: Detailed description of the ADL+ toolkit and screenshots; Table S2: Linear mixed-effects models for neuropsychological, activity levels, and quality of life outcomes at 6 and 9 months; Table S3: Adherence rate for the overall ADL+ toolkit and components.
