# Supplementary material for: ADL+: A Digital Toolkit for Multidomain Cognitive, Physical, and Nutritional Interventions to Prevent Cognitive Decline in Community-Dwelling Older Adults
Source: Int J Environ Res Public Health. 2024 Dec 31;22(1):42. doi: 10.3390/ijerph22010042 (PMC11764548; doi:10.3390/ijerph22010042)
Supplement: Supplementary file 1 [file ijerph-22-00042-s001.zip › ijerph-3305701-supplementary.pdf]

ADL+: a digital toolkit for cognitive assessment and multidomain intervention for prevention of cognitive decline in community-dwelling older adults

Supplementary Materials

S1. Detailed description of the ADL+ toolkit and screenshots

1. Smart Day Activity

The toolkit's central user interface is the Smart Day Activity module, an intelligent scheduling and advisory system designed to guide users in effectively navigating the ADL+ toolkit and coordinating their interactions with other modules. It employs a smart scheduler which is based on the Predictive Model and a Preference Structure. The Predictive Model supplies data concerning user conditions and requisite tasks, while the Preference Structure specifies the constraints related to activity plans and schedules as well as the preferences of users and community centres. The module has been implemented as a mobile application. Each day, the Smart Day Activity App generates a personalised task schedule for each user (see Figure S1(a)).

2. Cognitive Stimulation Module

The Cognitive Stimulation module, a smartphone game, fosters cognitive training by adapting the individual Cognitive Stimulation Therapy (iCST) concept for app-based delivery, eliminating the need for a therapist or caregiver. Customised to the Singapore context, the module offers 75 sessions across 21 themes. Encompassing diverse activities, the Cognitive Stimulation module includes visual and auditory puzzles, general knowledge and current affairs quizzes, social and physical activities, and open-ended questions.

ADL+: a digital toolkit for cognitive assessment and multidomain intervention for prevention of cognitive decline in community-dwelling older adults

Figure S1. Screenshots of the Smart Day Activity App: (a) schedule page; (b) completing tasks to accumulate rewards; screenshots of the Cognitive Stimulation module: (c) English version: auditory puzzle; (d) Chinese version: word puzzle

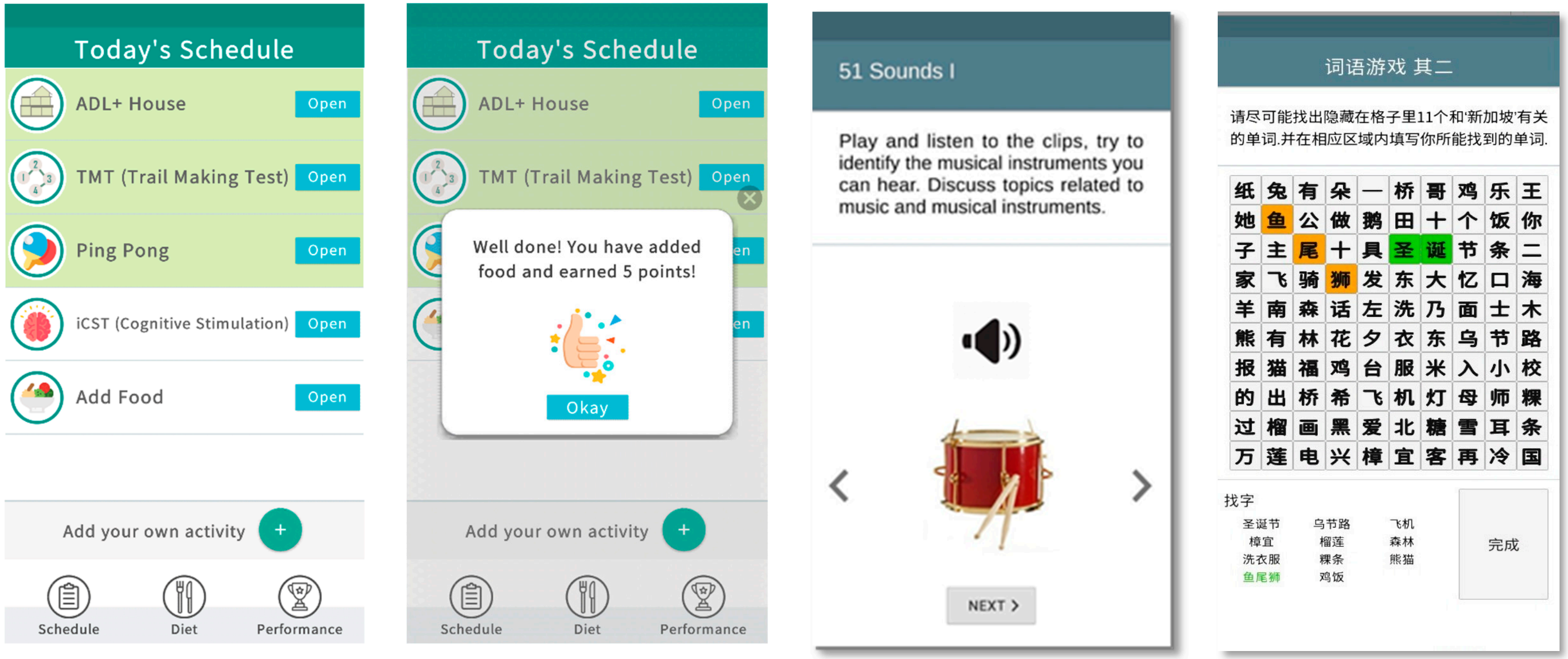

(a) (b) (c) (d)

3. Virtual ADL+ House Module

The Virtual ADL+ House module is a virtual home environment that promotes cognitive assessment and training through eight scenarios involving Instrumental Activities of Daily Living (IADL). Each scenario begins with a self-assessment based on the Lawton IADL scale, and specific to the IADL integrated in that scenario. Subsequently, it transitions to an immersive activity featuring a gamified cognitive test embedded within the IADL task. The

collection of scenarios within the Virtual ADL+ House incorporates nine gamified cognitive tests spanning multiple cognitive domains such as attention, processing speed, visuospatial skills, language, memory, and executive functioning. Throughout these game sessions, user performance and behavioural metrics, including time between taps, completion time, accuracy, backtracking instances, and unsubmitted or discarded responses, are recorded. This collected data is subsequently uploaded to a central database for utilisation by the Predictive Model module.

Figure S2. Screenshots of the Virtual ADL+ House

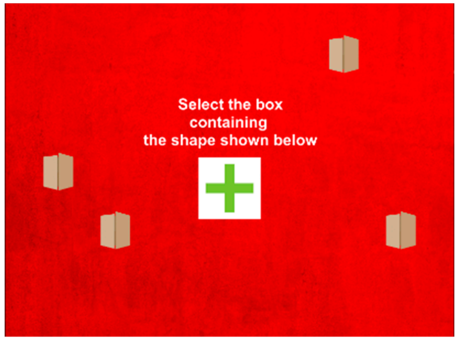

(a) Shopping Scenario
This scenario, based on the Paired Association Learning Test, involves finding patterns in a randomised sequence of boxes in a virtual warehouse. Users are shown a pattern, then contents of boxes one by one. Following this, the contents of the boxes are hidden, and users need to identify the box with the given pattern.

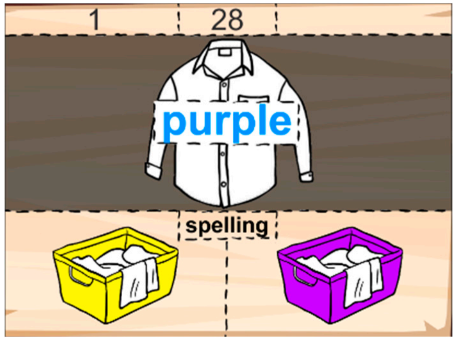

(b) Laundry Scenario
Based on the Stroop Test, this scenario requires sorting virtual laundry by colour. Each item has a colour-named tag, which may not match its font colour. In the first mode, users sort based on the tag's meaning. In the second, they sort based on the tag's font colour. The final mode combines both of these, requiring a perceptual set switch.

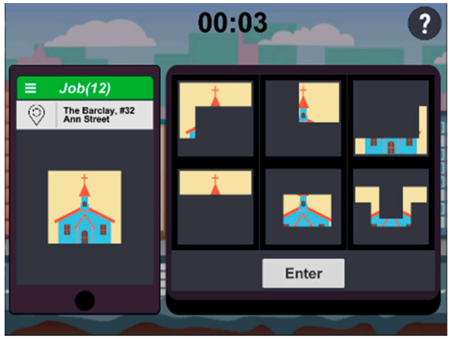

(c) Transportation Scenario
Designed based on the Visual Spatial Imagery Test, users need to assemble a visual puzzle of a landmark by selecting the correct three pieces.

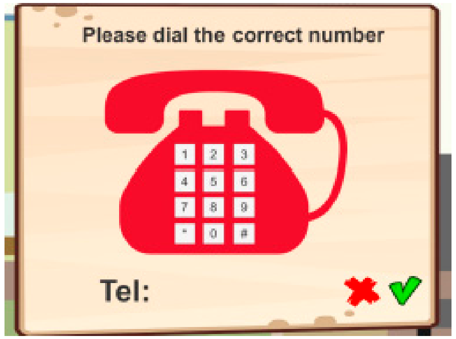

(d) Telephone Scenario
Based on the Digit Span Forward Test, this scenario presents a phonebook with numbers. After pressing "Make Call", users are moved to a phone interface where they have to remember and input the numbers shown previously.

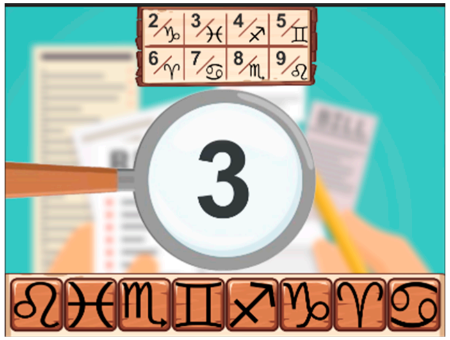

(e) Finance Scenario
Designed based on the Digit Symbol Modality Test, this scenario involves bill payment using unfamiliar symbols as "foreign languages". Given a symbol-number key, users encode the numbers on bills using corresponding symbols to make payments.

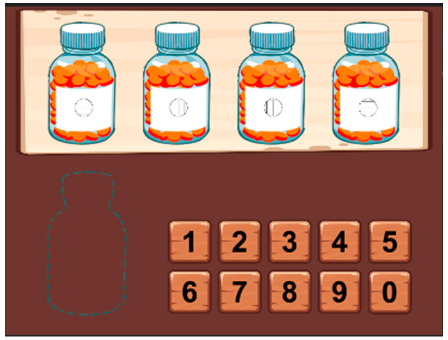

(f) Medication Scenario
Inspired by the Benton Visual Retention Test, this scenario involves managing medication, each represented by a geometrically labelled pill bottle. After a learning phase, users must identify the correct pill bottle from a list, recognising patterns on tags, and choose the right dosage.

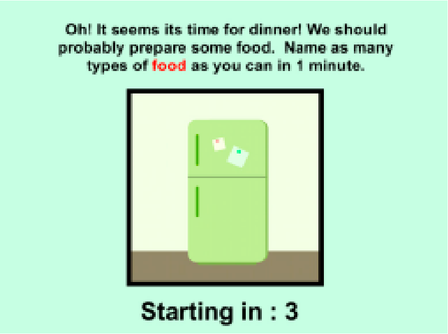

(g) Chores Scenario
Based on the Semantic Word Fluency Test, this scenario asks users to name as many items as possible in given categories such as "food" and "clothing", within a time limit.

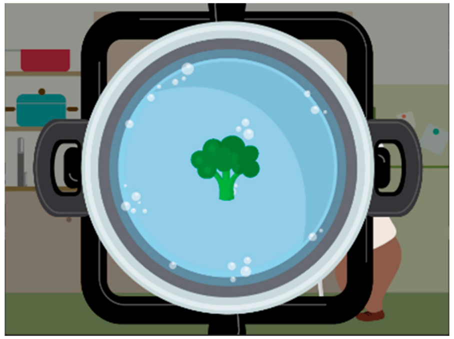

(h) Cooking Scenario
Incorporating the Simple Reaction Task and Motor Screening Test, this scenario has two modes. The first requires quick taps on vegetables in a virtual pot to avoid overcooking. In the second, users must tap only a specific vegetable type.

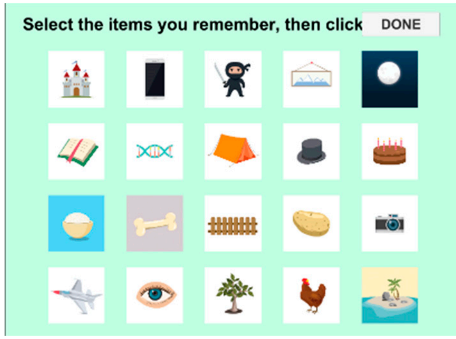

(i) Delayed Recall
Drawing from the CERAD Word List Memory Test, this scenario involves a memorisation phase and two recognition phases. Users memorise ten items, then identify them from a larger pool both immediately after viewing, and after a delay. The immediate phase repeats three times, while the delayed phase takes place after 15 min.

4. Online Trail Making Test

This digital version of the Trail Making Test (TMT) includes ten levels and a bonus level for cognitive training. In each level, users are required to memorise, identify, and connect a series of coloured, numbered dots from among a randomly generated pool. The bonus level is based on the Shape Trail (based on a TMT Part B variant), and assesses visuospatial ability, working memory, and task-switching by requiring users to connect dots in alternating shapes.

5. Physio-Cognitive Ping Pong

This Kinect-based table tennis game combines physical and cognitive exercise through dual-tasking. Users answer questions during play using a swinging motion, with on-screen prompts and response-tagged balls. Correct answers are scored by hitting back the right ball. A multiplayer mode also encourages social interaction.

Figure S2. Screenshots of Online TMT module: (a) instruction screen of a normal level; (b) game screen of a normal level

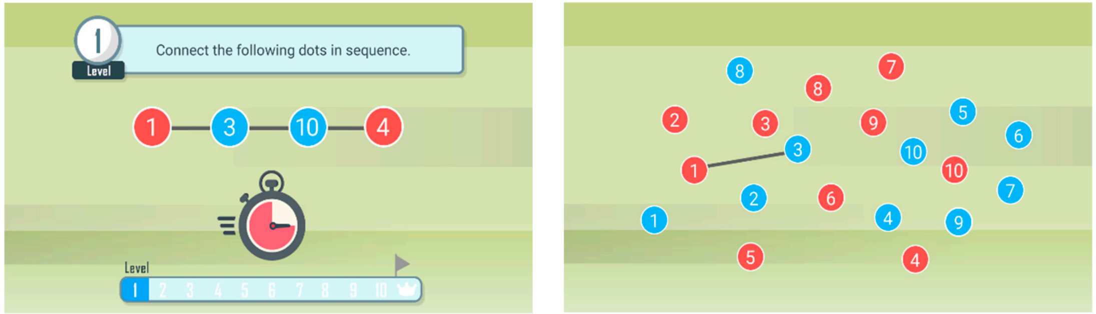

(a) **(b)**

Figure S3. Screenshots of the Physio-cognitive Ping Pong module: (a) interface displaying the question; (b) interface for hitting back the ball with correct answer

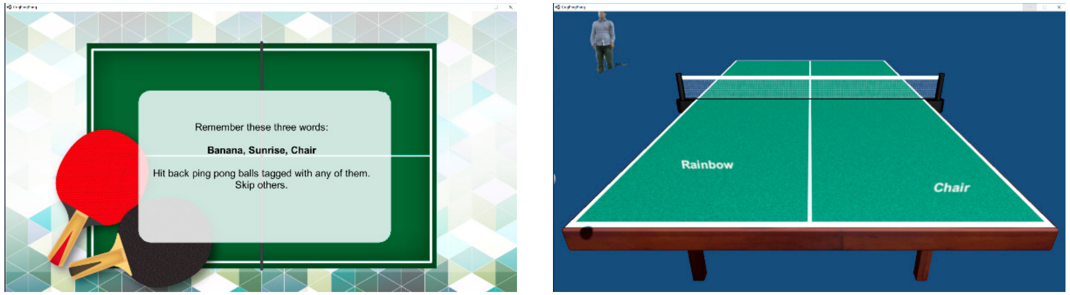

(a) **(b)**

6. Diet Analysis and Recommendation

The Diet Analysis module is an image-based application designed to log and analyse users' dietary intake. Utilizing advanced artificial intelligence and computer vision algorithms, this module enables users to capture photographs of their meals, which the system then analyses to identify the food items and assess nutritional content.

In Singapore, the richly diverse culinary culture presents a unique challenge: local cuisines are often not represented in publicly available food image datasets, which limits the accuracy of food recognition algorithms trained on these datasets. To address this, we have constructed a comprehensive local food dataset, incorporating over 500 distinct dishes and 140,000 images, crawled from web images and manually annotated to ensure quality. This dataset not only enhances our model's accuracy but also serves as a valuable resource for further research in local cuisine recognition.

We have developed a Deep Convolutional Neural Network (CNN) model for recognizing food from images. Our CNN model was trained intensively on both popular existing food datasets and our own local food datasets. The trained model achieved both high accuracy and small losses. Our model can locate important areas that contain food features in an image and distinguish these areas from messy backgrounds (as shown in Figure S1). Experiment results illustrate that the top-5 recognition accuracy is over 87%. Our model has demonstrated great robustness to the dishes with rigid shapes, such as burgers, egg

tarts, fried chicken wings, etc. In real applications, the users snap a photo of their meals with mobile phones and then the inference server will analyse the contents of images.

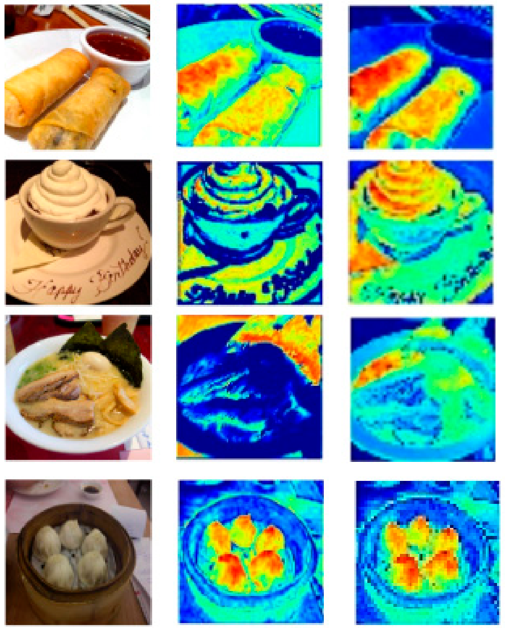

Figure S4 Food image recognition algorithm in Diet Analysis and Recommendation module - the areas highlighted in red and yellow are the important areas with food features identified by our Deep Convolutional Neural Network (CNN) model

To accurately assess the nutritional content of foods identified from images, we developed a specialised nutrition database. This database was constructed using data from the Health Promotion Board's website[1], which details the energy and nutrient composition of various foods. Using this information, we extracted nutritional components for different recipes. Once a food item is recognized from a user-uploaded image, our module queries this nutrition database to retrieve corresponding recipes and nutritional data. It then automatically populates the nutritional values for the recognized food items (as shown in 5(a)). Users have the option to overwrite these default values. Updating fields such as portion size will also result in an automatic update of the nutrition values. The system records and analyses this nutrition data to generate intelligent nutrition summaries (see **Error! Reference source not found.**(c)). These summaries provide insights into the nutritional contributions of each food item, compare current nutrient intake levels with Recommended Dietary Allowances (RDA) values, and highlight dietary imbalances. By visualizing how current consumption aligns with recommended standards, this intelligent analysis serves as a tool to guide users towards healthier dietary choices.

Figure S5. Screenshots of the Diet Analysis and Recommendation module: (a) Add food with nutritional values – users can select from provided nutritional values or input their own; (b) Food diary displaying logged meals; (c) Nutrient analysis showing carbohydrate intake

[1] Energy and Nutrient Composition of Food, Health Promotion Board: https://focos.hpb.gov.sg/eservices/ENCF/

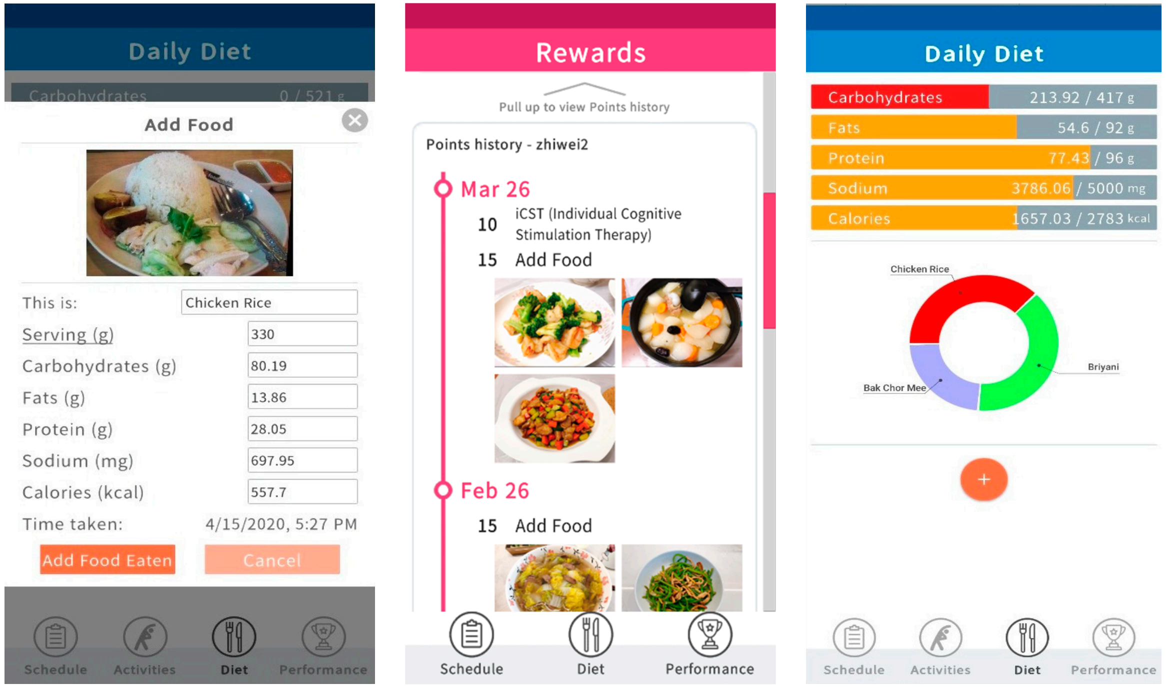

(a) (b) (c)

7. Predictive Model

The Predictive Model module utilises data from other modules to assess an individual's cognitive functioning. It takes in nutrition data from the Diet Analysis and Recommendation module, as well as game performance data from the four assessment modules. Through the utilisation of multiple primary components, each corresponding to a specific cognitive aspect, the Predictive Model generates predictions for individual users. These primary components encompass processing speed, attention, language, visuospatial skills, memory, and executive functioning.

Table S2: Linear mixed effects models for neuropsychological, activity levels and quality of life outcomes at 6 and 9 months

Variable	Intervention	Control	Difference (95% CI)**	P
	Mean (95% CI)	Mean (95% CI)		
**Change in NTB domain and composite scores***				
**NTB Composite**<br>**(Attention, Processing Speed, Memory, Executive Function)**				
At 6 months	0.086 (0.020-0.15)	-0.087 (-0.16- -0.013)	0.17 (0.071-0.27)	0.001
At 9 months	0.016 (-0.065 – 0.096)	-0.023 (-0.11-0.068)	0.038 (-0.085-0.16)	0.54
**Attention**				
At 6 months	0.15 (0.002-0.29)	-0.13 (-0.28-0.03)	0.2 (0.055-0.49)	0.014
At 9 months	-0.0016 (-0.16-0.16)	-0.055 (-0.23-0.12)	0.053 (-0.19-0.25)	0.66
**Processing speed**				
At 6 months	0.070 (-0.038-0.18)	-0.17 (-0.28-0.18)	0.24 (0.073-0.40)	0.004
At 9 months	0.054 (-0.049-0.16)	-0.16 (-0.27- -0.050)	0.21 (0.059-0.37)	0.007
**Memory**				

At 6 months	0.092 (-0.028-0.21)	-0.11 (-0.24-0.017)	0.20 (0.021-0.38)	0.029
At 9 months	0.014 (-0.13-0.16)	-0.064 (-0.22-0.087)	-0.0097 (-0.18-0.16)	0.47
**Executive function**				
At 6 months	0.097 (-0.017-0.21)	-0.050 (-0.17-0.074)	0.15 (-0.025-0.32)	0.095
At 9 months	0.028 (-0.10-0.16)	0.014 (-0.13-0.15)	0.014 (-0.18-0.21)	0.89
**mCMMSE**				
At 6 months	-0.036 (-0.42-0.35)	-0.47 (-0.87- -0.063)	0.43 (-0.14-1.00)	0.14
At 9 months	-0.23 (-0.86-0.22)	-0.75 (-1.22- -0.28)	0.52 (-0.14-1.18)	0.13
**FAI**				
At 6 months	0.40 (-0.35-1.15)	-1.04 (-1.83- -0.26)	1.44 (0.33-2.56)	0.011
At 9 months	-1.08 (-2.04- -0.11)	-1.79 (-2.78- - 0.79)	0.71 (-0.70-2.12)	0.32
**EQ-5D utility scores**				
At 6 months	0.041 (0.016-0.066)	0.033 (0.0062-0.059)	0.0080 (-0.029-0.046)	0.68
At 9 months	0.039 (0.0060-0.071)	0.039 (0.005-0.073)	-0.00027 (-0.048-0.047)	0.99

**EQ-5D VAS**				
At 6 months	4.46 (1.89-7.04)	0.40 (-2.31-3.11)	4.06 (0.23-7.90)	0.038
At 9 months	5.46 (2.52-8.40)	0.34 (-2.71-3.38)	5.12 (0.81-9.43)	0.020

NTB: Neuropsychological test battery; mCMMSE: modified version of the Chinese Mini-Mental State Examination; FAI: Frenchay Activities Index; EQ-5D: EuroQoL-5 Dimensions; VAS: visual analogue scale

*Mean changes from baseline were estimated using repeated-measures linear mixed-effects models. In addition to trial group, time and the interaction of trial group with time, the model included the respective baseline scores, age, gender, education level and baseline GDS scores as covariates.

**Difference refers to intervention – control, shown for mean changes from baseline

ADL+: a digital toolkit for cognitive assessment and multidomain intervention for prevention of cognitive decline in community-dwelling older adults

Table S3. Adherence rate for the overall ADL+ toolkit and components

Module	Median adherence (%)	IQR
Overall ADL+ Toolkit	95.0	88.3-98.3
Virtual ADL+ House	94.4	88.2-100
Online TMT	100.0	94.1-100
Physio-Cognitive Ping Pong	97.7	93.3-100
iCST Games	98.3	94.4-100
Diet logging	95.3	87.2-98.3
Smart Day Activities	94.2	84.6-100

TMT: Trail Making Test; iCST: Individual Cognitive Stimulation Therapy
